# Wound microenvironment-responsive glucose consumption and hydrogen peroxide generation synergistic with azithromycin for diabetic wounds healing

**DOI:** 10.7150/thno.64244

**Published:** 2022-03-06

**Authors:** Minqi Shi, Zhen Du, Yuchen Qi, Wanlin Li, Huiqun Hu, Xiuhui Lin, Shoujie Wang, Zhe Tang, Min Zhou

**Affiliations:** 1 Department of Surgery, The Fourth Affiliated Hospital, Zhejiang University School of Medicine, Yiwu 322000, China.; 2 Institute of Translational Medicine, Zhejiang University, Hangzhou 310009, China.; 3 The Cancer Hospital of the University of Chinese Academy of Sciences (Zhejiang Cancer Hospital), Institute of Basic Medicine and Cancer (IBMC), Chinese Academy of Sciences, Hangzhou, Zhejiang 310022, China.; 4 State Key Laboratory of Modern Optical Instrumentations, Zhejiang University, Hangzhou 310058, China.; 5 Department of Infectious Diseases, The Second Affiliated Hospital, Zhejiang University School of Medicine, Hangzhou 310009, China.

**Keywords:** silica nanoparticle, glucose oxidase, azithromycin, bacterial biofilm, diabetic wound healing

## Abstract

**Rationale:** Chronic wounds are one of the common complications of diabetes. Due to the physiological conditions of diabetic patients, these wounds are more susceptible to bacterial infections and the formation of bacterial biofilms, leading to the inefficiency of conventional antibiotic treatment.

**Methods:** Here, hollow mesoporous silica nanoparticles (HMSN) were used as the nanocarriers for co-delivery of azithromycin (AZM) and glucose oxidase (GOX), achieving a remarkable synergistic effect in chronic diabetic wounds. GOX possesses the catalytic ability to consume glucose and produce H_2_O_2_ in the diabetic wound area. The down-regulation of local glucose could effectively improve the chronic diabetic wound microenvironment. Meanwhile, the generated H_2_O_2_ effectively inhibits bacterial growth and eradicates bacterial biofilms with the synergism of antibiotics AZM.

**Results:** In the bacteria-infected diabetic cutaneous wound models, the reduction of glucose, generation of H_2_O_2_, and release of AZM could effectively reduce the bacterial infection and promote the wounds healing. Moreover, there is no obvious toxicity behavior after the treatment.

**Conclusions:** Therefore, the designed nanosystem could effectively accelerate the diabetic wound healing process by the amelioration of the hyperglycemia microenvironment and the eradication of bacterial biofilms around the wounds, making them promising candidates for clinical transformation.

## Introduction

Chronic wounds are one of the major complications associated with diabetes, which represent a huge burden for both affected individuals and the entire healthcare system 1,2. Diabetic patients have declined ability to metabolize glucose resulting in hyperglycemic conditions which further prolong the wound healing process 3-5. The reasons for poor wounds healing in chronic diabetes include abnormal fibroblasts activity, peripheral neuropathies, low vascularization and high microbial burden 6-9. Among them, bacterial infection tends to be a hazard, which leads to the recurrent and persistent local inflammation of the wounds 10-12. And what is worse, the hyperglycemia microenvironment of diabetic wounds is an ideal breeding ground for bacteria colonization and biofilm formation, which makes wound healing more difficult 13-15. Biofilms are surface-associated communities of microorganisms encased in a self-produced polymeric matrix, which are known as extracellular polymeric substances (EPS) 16-18. The EPS protects bacteria against desiccation, oxidizing, ultraviolet radiation and some antibiotics 19-22. It is reported that hyperglycemia promotes the formation and the expansion of biofilms through creating a surplus nutrient source for bacteria, leading to enhanced tolerance to antibiotics and the failure of traditional antibiotic treatments 23,24. Therefore, reducing local glucose concentration in diabetic wounds is expected to inhibit wound infection and enhance the therapeutic effect of antibiotic drugs.

Glucose oxidase (GOX) is an endogenous oxidoreductase that can specially catalyze glucose oxidation into gluconic acid and hydrogen peroxide (H_2_O_2_) with the assistance of oxygen 25-28. Over recent years, GOX has aroused great research interest in the biomedical field due to its inherent biocompatibility, non-toxicity and unique catalytic properties against glucose 29-34. Therefore, GOX is expected to reduce local glucose concentration in diabetic wounds through catalytic reactions and improve the therapeutic effect of antibiotics. It is worth noting that GOX can not only catalyze the degradation of glucose, but also produce a strong oxidizing free radical, H_2_O_2_. It is reported that H_2_O_2_ along with its secondary metabolite hydroxyl radical could damage bacteria cell components, leading to the breakdown of bacteria cell membranes and walls 35,36. Additionally, H_2_O_2_ promotes wound healing by mediating leukocyte recruitment, facilitates the release of vascular endothelial growth factor and improved blood flow 37-39. Taken together, it is considered that GOX plays two important roles in accelerating the wound healing process of diabetic wounds: (i) reducing the local glucose concentration. (ii) producing H_2_O_2_. Therefore, GOX is expected to ameliorate the conditions of diabetic wounds and enhance the therapeutic efficacy of antibiotics.

Topical antibiotic application is fundamental for the treatment of skin infections 40. Azithromycin (AZM) is a macrolide antibiotic usually used against gram-positive bacteria 41. It inhibits protein synthesis by binding reversibly to 50s ribosomal subunits of sensitive bacteria 42-44. However, due to the antibiotic resistance of biofilms, the efficient treatment of wounds especially diabetic chronic wounds is still an immediate and formidable challenge. Fortunately, GOX effectively reduces local glucose concentration and produces free radicals to destroy the growth environment of biofilms, which is expected to promote the antibacterial effect of AZM.

Inspired by this, we developed GOX and AZM dual-functionalized hollow mesoporous silica nanoparticles (GOX-HMSN-AZM) that possess antibacterial effects and local glucose reduction simultaneously to facilitate bacteria-infected diabetic wound healing. Due to the special spatial structure, high specific surface area and modification sites of HMSN, it enables both the effective loading of AZM into its internal cavity and the GOX functionalization of the material surface. On the one hand, GOX can react with glucose to produce H_2_O_2_, which effectively improved the local environment of diabetic wounds so as to inhibit bacterial biofilm and accelerate the wound healing process. On the other hand, AZM as an antibiotic effectively eradicated bacteria by inhibiting protein synthesis. GOX and AZM exhibited synergistic effects to realize the effective regulation of diabetic wounds environment, anti-infection, and ultimately promote wound healing of bacteria-infected diabetic mice.

## Materials and Methods

### Materials

Glucose oxidase (GOX) and (3-Aminopropyl) triethoxysilane (APTES, ≥98%) were purchased from Sigma. Azithromycin (AZM, 98%) and triethanolamine (TEA, 98%) were purchased from Macklin (Shanghai, China). Tetraethyl orthosilicate (TEOS, >99%) and hexadecyltrimethylammonium chloride (CTAC, 97%) were purchased from Aladdin. Staphylococcus aureus (*S. aureus*, ATCC 6538) was obtained from the American type culture collection (Rockville, MD). Phosphate-buffered saline (PBS, 1X, pH = 7.2 ± 0.1) was purchased from Cienry (Zhejiang, China). Glucose, Luria-Bertani (LB) broth and LB broth agar were purchased from Sangon Biotech (Shanghai, China). Live/Dead BacLight bacterial viability kit was purchased from Thermo Fisher Scientific. Db/db mice were obtained from SLAC Laboratory Animal Co., Ltd (Shanghai, China). Purified water was used throughout.

### Synthesis of HMSN

Firstly, dense SiO_2_ (dSiO_2_) nanoparticles were prepared according to a modified Stöber method. The mixed solution of 142.8 mL ethanol, 20 mL water and 3.2 mL ammonia were stirred for 10 minutes at room temperature, followed by the addition of 4 mL TEOS. Then the mixture was stirred for 1h. dSiO_2_ nanoparticles were collected by centrifugation, washed with water twice and suspended in 40 mL water. Secondly, the synthesis of dSiO_2_@MSN. 16 g CTAC and 160 mg TEA were mixed in 200 mL water and stirred at 50 ºC. After mixing completely, 40 mL dSiO_2_ solution was added and stirred for 1 h at 80 ºC. Then 1.2 mL TEOS was dropwise added and kept stirring for 1 h. Thirdly, HMSN was obtained by etching dSiO_2_@MSN 45. After cooling down to 50 ºC, 5.1 g Na_2_CO_3_ was used to selectively etch the internal dSiO_2_ core and the mixture was stirred for 30 min. The resulting HMSN were collected by centrifugation (12000 rpm, 10 min) and then were extracted with a methanol solution of NaCl (1 wt %) for 24 h at room temperature three times to remove CTAC. Finally, the HMSN were dispersed in the water.

### Synthesis of HMSN-AZM

To load AZM into the cavity of HMSN, 50 mg HMSN and the appropriate amount of AZM were dissolved in ethanol and stirred for 24 h at room temperature. The resulting HMSN-AZM were collected by centrifugation (12000 rpm, 10 min) and washed with water. The standard solution of AZM was prepared for UV-vis determination to construct a standard curve. AZM loading capacity was obtained by measuring the AZM concentration of the supernatant before and after loading.

### Synthesis of GOX-HMSN-AZM

GOX was functionalized onto the surface of HMSN by covalent grafting according to the literature method 31. In brief, 95 mg EDC, 142.5 mg NHS and 10 mg GOX were dissolved in 15 mL water, followed by the addition of 112 µL APTES. The mixture was stirred for 8 h at room temperature to produce amino-functionalized GOX. Then 50 mg HMSN-AZM was added to the mixture and stirred for 24 h. The final nanoparticles (GOX-HMSN-AZM) were collected by centrifugation and washed with water three times.

### Synthesis of GOX-HMSN

95 mg EDC, 142.5 mg NHS and 10 mg GOX were dissolved in 15 mL water, followed by the addition of 112 µL APTES. The mixture was stirred for 8 h at room temperature to produce amino-functionalized GOX. Then 50 mg HMSN was added to the mixture and stirred for 24 h. The final nanoparticles (GOX-HMSN) were collected by centrifugation (12000 rpm, 10 min) and washed with water three times.

### Characterization

The morphology of the nanoparticle and bacteria were characterized by Transmission electron microscopy (FEI, Tecnai F20, USA). Hydrodynamic size and zeta potential measurements were performed at room temperature by a dynamic light scattering system (Malvern Panalytical, Zetasizer Nano ZS90, UK). XRD patterns were characterized by the X-ray diffractometer (Panalytical X'PERT PRO, Netherlands). Raman spectra were obtained by Raman spectrometer (Renishaw, InVia Reflex, UK). UV-Vis spectra were recorded with an ultraviolet spectrophotometer (Shimadzu, UV-2600, Japan). FTIR patterns were characterized on an FTIR Spectrometer (Madison, Nicolet Nexus 470, USA).

### *In vitro* Measurement of Glucose

20 mM glucose solution was prepared, which was co-cultured with different concentrations (0, 10, 25, 50, 100, 200, 400 μg/mL) of GOX-HMSN or GOX-HMSN-AZM at 37 ºC for 12 h. Then, a glucometer (Yuyue, 580, China) was used to measure the glucose concentration of the different groups. Besides, 20 mM glucose solution was cultured with 200 μg/mL of GOX-HMSN or GOX-HMSN-AZM at 37 ºC. The glucose concentration at different incubating times (0, 1, 3, 6, 12, 24 h) were recorded using a glucometer (n = 3).

### *In vitro* Measurement of H_2_O_2_ Concentration

Firstly, 1.33 mL of 24% Ti(SO_4_)_2_ and 8.33 mL of H_2_SO_4_ were added in 50 mL distilled water to prepare the Ti(SO_4_)_2_ cocktail detection solution. The standard solutions of glucose were mixed with Ti(SO_4_)_2_ cocktail detection solutions (1:1), and the corresponding UV-vis absorptions at 405 nm were recorded to construct an H_2_O_2_ standard curve. Then, 20 mM glucose solution was prepared, which was cultured with different concentrations (0, 10, 25, 50, 100, 200, 400 μg/mL) of GOX-HMSN or GOX-HMSN-AZM at 37 ºC for different times. The supernatant was collected by centrifugation (12000 rpm, 10min) and then was mixed with Ti(SO_4_)_2_ cocktail detection solution (1:1). The absorbance was measured at 405 nm and then the concentration of H_2_O_2_ was acquired by the standard curve (n = 3).

### *In vitro* Measurement of pH

20 mM glucose solution was prepared, which was cultured with different concentrations (0, 10, 25, 50, 100, 200, 400 μg/mL) of GOX-HMSN or GOX-HMSN-AZM at 37 ºC for 12 h. Afterwards, a pH meter (Mettler Toledo, FiveEasy Plus, Switzerland) was used to measure the pH values. Besides, 20 mM glucose solution was cultured with 200 μg/mL of GOX-HMSN or GOX-HMSN-AZM at 37 ºC. Using the pH meter to measure the pH variation at different incubating times (0, 1, 3, 6, 12, 24 h) (n = 3).

### *In vitro* drug release study

The standard solution of AZM was prepared and the corresponding UV-vis absorptions at 215 nm were recorded to construct a standard curve. The HMSN-AZM and GOX-HMSN-AZM were incubated with glucose (20 mM) at 37 ºC. At predetermined time points (0, 1, 3, 6, 12 and 24 h), the supernatant of the solution was collected by centrifugation (12000 rpm, 10min) and then evaporated. Ethanol was used to dissolve the released AZM. The absorbance was measured and then the concentration of AZM was acquired by the standard curve (n = 3).

### Measurement of the capacity of GOX

The concentration of GOX was determined using a BCA protein assay kit. Protein standards (0、1、2、4、8、12、16、20 μL), GOX-HMSN (20 μL) and GOX-HMSN-AZM (20 μL) were added into 96-wells plates (n=3). Then 200 μL working reagent was added to each well and mixed. The solution was incubated at 60 ºC for 30 min. A multimode microplate reader was used to measure the optical density at 562 nm. Then the standard curve of protein was prepared and the GOX concentration was determined by the standard curve (n = 3).

### Enzyme Activity Assay

For the enzyme kinetic measurements of the nanocomposites, 0-30 mM glucose was used as substrate. GOX-HMSN and GOX-HMSN-AZM were incubated with different concentrations of glucose at 37 ºC for 30 min. The supernatant was collected by centrifugation (12000 rpm, 10 min). Then the Ti(SO_4_)_2_ cocktail detection solution was added to measure the production of H_2_O_2_. The absorbance was measured at 405 nm and then the concentration of H_2_O_2_ was acquired by the standard curve. The data were analyzed using the Lineweaver-Burk plot and the kinetic parameters K_m_ and K_cat_ were calculated using the Michaelis-Menten Equation (n = 3).

### Storage Stability of Enzymes

GOX-HMSN and GOX-HMSN-AZM nanoparticles were stored at 4 ºC. After 0, 3, 7, 14 and 30 days, 20 mM glucose solution was mixed with the nanoparticles to initiate the reaction. The supernatant was collected by centrifugation (12000 rpm, 10min) and then was mixed with Ti(SO_4_)_2_ cocktail detection solution (1:1). The absorbance was measured at 405 nm and the concentration of H_2_O_2_ was acquired by the standard curve (n = 3).

### *In vitro* Antibacterial Activity Analysis

*S. aureus* suspension (10^9^ CFU/mL) was cultured with different concentrations (0, 3.91, 7.81, 15.63, 31.25, 62.50 μg/mL) of HMSN, HMSN-AZM, GOX-HMSN and GOX-HMSN-AZM in LB broth (20 mM Glucose contained) for 24 h at 37 ºC. After incubation, a multimode microplate reader (Molecular Devices SpectraMax, M5, USA) was used to measure the bacteria concentration by determining the optical density at 600 nm (n = 4).

### Bacterial Resistance Test

A minimum inhibitory concentration (MIC) test was used to evaluate the bacterial resistance. Firstly, *S. aureus* suspension (10^9^ CFU/mL, passage 1) was cultured with different concentrations of AZM and GOX-HMSN-AZM in LB broth (20 mM Glucose contained) for 24 h at 37 ºC. Then the bacteria survived after half MIC (passage 1) treatment were designated as passage 2. The above operations were repeated for 10 successive passages (n=3).

### Bacterial Live/Dead Fluorescent Assay

*S. aureus* suspension (10^9^ CFU/mL) was incubated with HMSN, HMSN-AZM, GOX-MSN and GOX-HMSN-AZM at 15.6 μg/mL for 3 h at 37 ºC. Then SYTO 9/PI live/dead bacteria viability kit was used following the corresponding instruction. Briefly, SYTO 9 and PI were diluted with PBS (1:1000) and then mixed with the treated bacterial suspension above for 20 min at room temperature. After that, the bacterial suspension was placed on glass slides. Images were obtained with a fluorescence confocal microscope (Leica, TCS SP8, Germany).

### Morphological Characterization of Bacteria

*S. aureus* suspension was diluted to 10^9^ CFU/mL, then was cultured with 15.6 μg/mL HMSN, HMSN-AZM, GOX-HMSN, GOX-HMSN-AZM for 6 h. The suspension was collected by centrifugation and fixed by glutaraldehyde solution (2.5%) for 12 h. Afterwards, the sample was washed three times with PBS for 15 min and embossed with agar. Then the sample was fixed by osmic acid for 2 h, followed by a gradient of dehydration with an ethanol-water mixture (30%, 50%, 70%, 90%, 95%, and 100%) for 10 min in turn. Finally, the dried sample was sputter-coated with gold and analyzed by TEM (FEI, Tecnai F20, USA).

### *In vitro* Antibacterial Effects Against Bacterial Biofilm

Biofilm formation was detected by the crystal violet method. Briefly, *S. aureus* suspension (10^7^ CFU/mL) was incubated in 96-wells plates with LB broth (20 mM glucose contained) for 24 h to form bacterial biofilms. Then the medium was replaced by fresh LB broth (20 mM glucose contained) with different concentrations (0, 62.5, 125, 250, 500, 1000 μg/mL) of HMSN, HMSN-AZM, GOX-HMSN and GOX-HMSN-AZM. After 24 h of incubation, the wells were washed by PBS for twice to remove free bacteria. Subsequently, the bacterial biofilms were stained with 100 μL crystal violet (1%) solution for 20 min at room temperature and then washed with PBS three times to remove excess dye. Then, 100 μL ethanol was added to each well and incubated for 10 min at room temperature. The optical densities of crystal violet were measured by the multimode microplate reader at 570 nm. In addition, the standard dilution plate assay was used to evaluate the effects against bacterial biofilms quantitatively. The bacterial biofilms were transferred into the centrifuge tube. After 10 min of ultrasonication, the living bacteria were obtained by gradient dilution and plate counting method (n = 3).

### Bacterial Biofilm Fluorescent Assay

*S. aureus* suspension (10^7^ CFU/mL) was incubated in 12-wells plates with LB broth (20 mM glucose contained) for 24 h to form bacterial biofilms. Then the medium was replaced by fresh LB broth (20 mM glucose contained) with 500 μg/mL HMSN, HMSN-AZM, GOX-HMSN and GOX-HMSN-AZM. After 24 h of incubation, the wells were washed by PBS for twice to remove free bacteria. Then SYTO 9 bacterial viability kit was used to stain bacterial biofilms. Briefly, SYTO 9 was diluted with PBS (1:1000) and then added 1 mL of SYTO 9 solution to each well for 20 min at room temperature. The biofilm morphology was examined under a 3D confocal scanning microscopy (Leica, TCS SP8, Germany) later.

### *In vivo* Treatment of Wounds

All animal experiments were approved by the institutional animal care and use committee of Zhejiang University (the animal experiment license number of the institution: 2020-844) and were individually raised in cages under standardized temperature. Db/db mice (female, 8 weeks old) were anesthetized by inhalation of isoflurane and shaved on the dorsum. Round skin wound injury (diameter of 10 mm) was generated with a surgical scalpel. Then the mice (n=3) were divided into different groups randomly. Skin wounds of all groups were infected by *S. aureus* suspension (10^7^ CFU/mL, 50 μL). After 24 h, mice were treated with HMSN, HMSN-AZM, GOX-HMSN and GOX-HMSN-AZM (500 μg/mL, 100 μL) respectively except that the control group was treated with PBS. Wound areas were photographed and measured at different times after treatments. Then the wound tissues were excised for pathological histology and immunohistochemistry analysis after 14 days of treatments. These tissues were washed with PBS and fixed in 10% formalin. Then the treated tissues were embedded in paraffin and sectioned into 5 μm sections. These sections were analyzed with hematoxylin and eosin (H&E), Masson's trichrome staining and immunohistochemistry method (VEGF, CD31 and FGF-2). All sections were examined by a virtual digital slide scanning system.

### Pilot Toxicity Study

After the above treatment of wounds (14 days), the blood samples were obtained for clinical chemistry and hematologic analysis. Then major organs (heart, lung, liver, spleen, and kidney) were harvested from mice in each group, washed with PBS and fixed in 10% formalin. Then the treated tissues were embedded in paraffin and sectioned into 5 μm sections. These sections were stained with H&E for toxicology histological analysis. All sections were examined by a virtual digital slide scanning system.

## Results and Discussion

### Synthesis and Characterization of GOX-HMSN-AZM

**Scheme 1:**
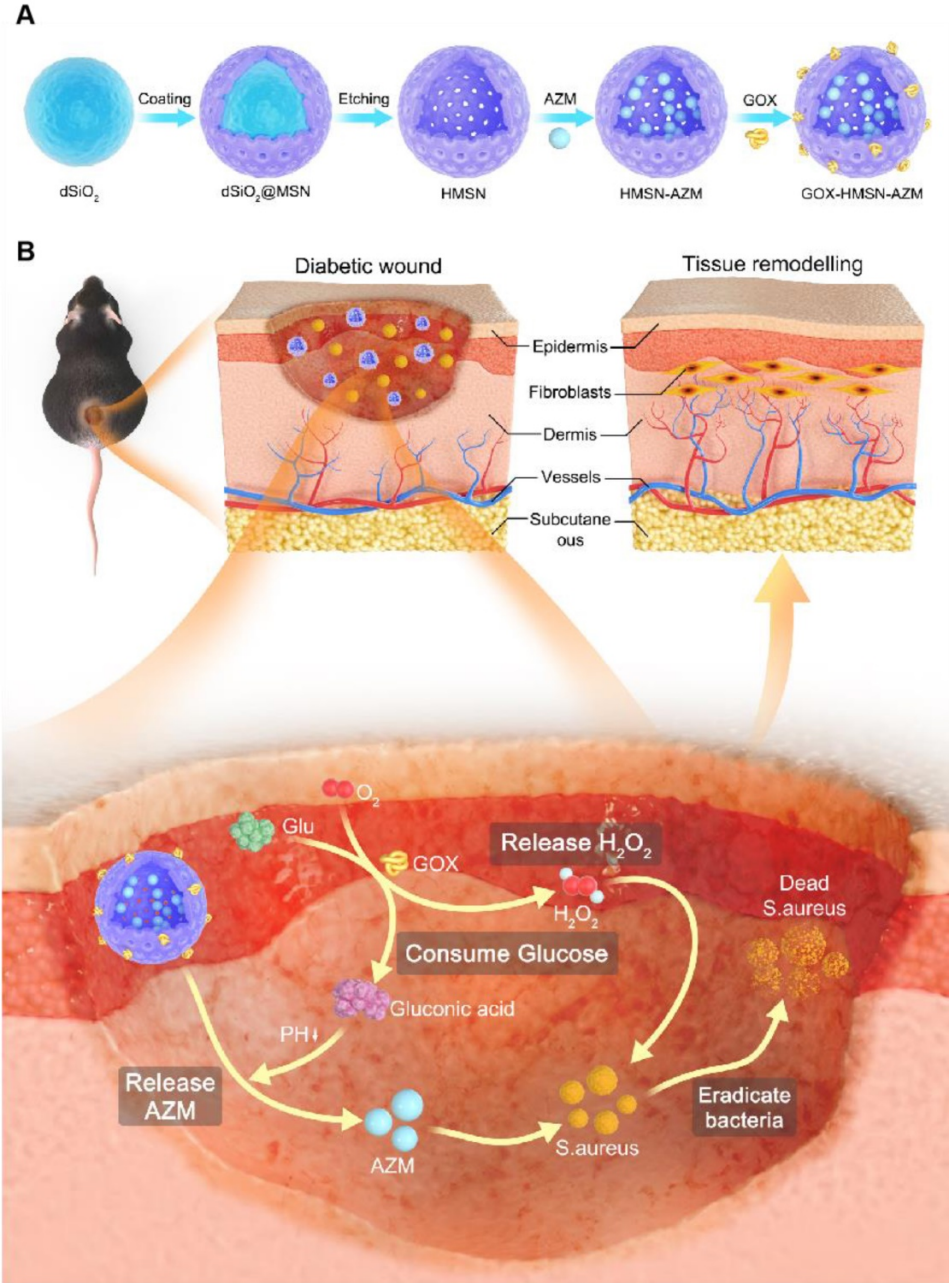
** Schematic illustration of the design and application of GOX-HMSN-AZM for diabetic wounds healing.** (A) The synthetic route of GOX-HMSN-AZM. (B) The schematic diagram of promoting the diabetic wounds healing with the treatment of GOX-HMSN-AZM.

It is well known that silicon nanoparticles have been widely employed as drug delivery platforms due to their excellent loading capacity, biocompatibility, and biodegradability 46-48. Here, we exploited silica nanoparticle with a special structure as the nanocarrier for the co-delivery of AZM and GOX. Hollow mesoporous silica nanoparticles (HMSN) were synthesized according to a selective etching strategy, and the synthetic route is illustrated in **Scheme 1A**. Firstly, uniform dense SiO_2_ (dSiO_2_) nanoparticles about 112 nm in size were synthesized as hard templates, which possessed negative surface charge (-47.0 mV) (**Figure S1A, Figure S2**). Secondly, the *in situ* coating of a mesoporous silica shell on and around dSiO_2_ nanoparticles was carried out (**Figure S1B, Figure S2**). Finally, dSiO_2_ and soft templates (CTAC) were selectively removed to generate the HMSN nanoparticles.

**Figure 1:**
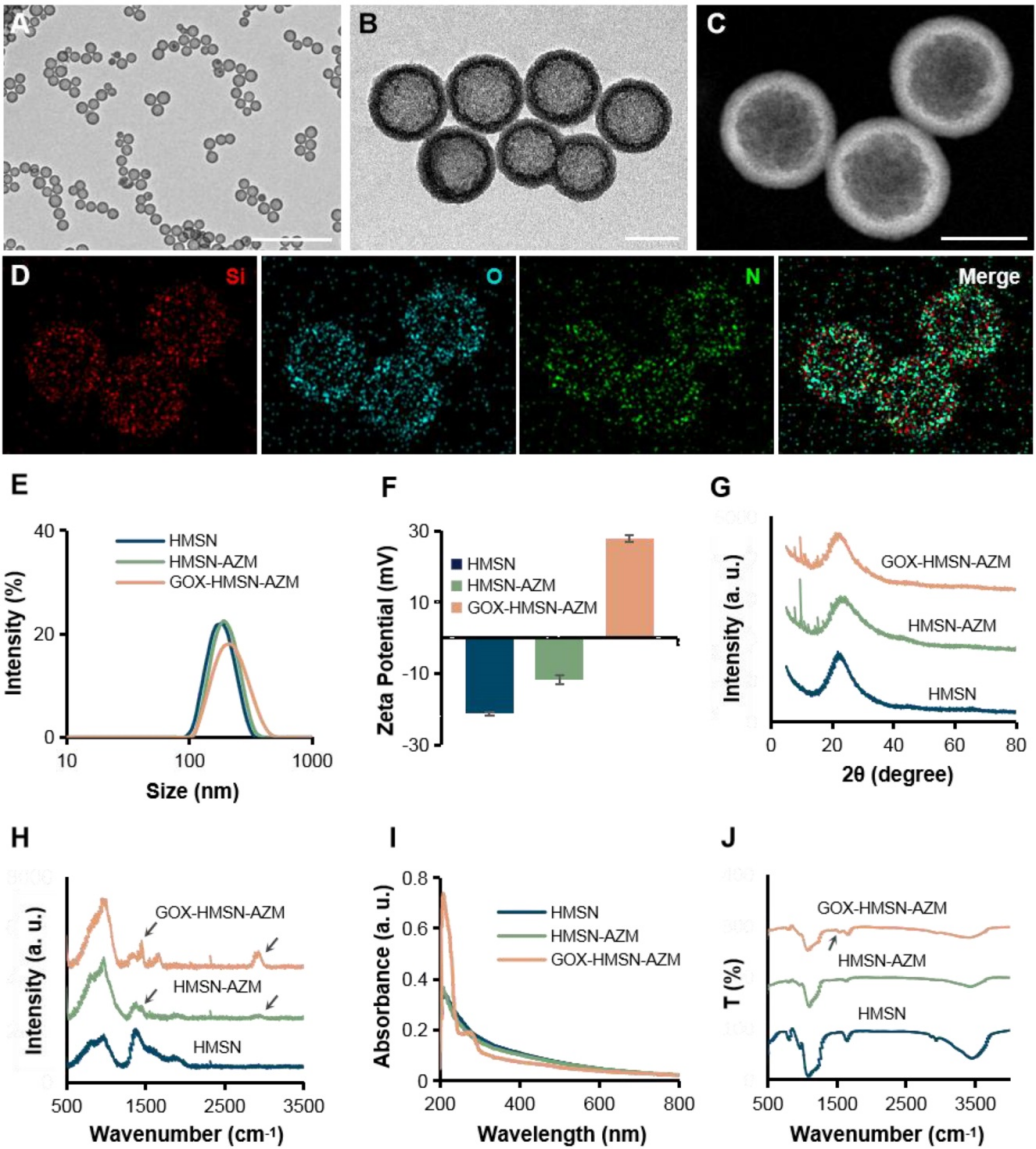
** Characterization of GOX-HMSN-AZM.** (A-B) Representative TEM images of HMSN and GOX-HMSN-AZM. Scale bar = 1μm and 100 nm, respectively. (C) The high-angle annular dark-field (HAADF) stem image of GOX-HMSN-AZM. (D) Elemental mapping (Si, O, N, Merge) of GOX-HMSN-AZM. (E) Hydrodynamic diameter distribution and (F) Zeta potential of HMSN, HMSN-AZM and GOX-HMSN-AZM measured by dynamic light, respectively. (G) XRD patterns, (H) Raman spectra, (I) UV-vis-NIR absorption spectrum, and (J) FTIR spectra of HMSN, HMSN-AZM and GOX-HMSN-AZM, respectively.

Transmission electron microscope (TEM) images in **Figure 1A** showed that the as-synthesized HMSN has a huge cavity, which makes it promise candidate for drug loading. Brunner-Emmet-Teller (BET) measurements show that HMSN has a relatively large surface area of 617 m^2^/g and a pore size of 3.76 nm (**Figure S3**), which facilitates efficient loading of guest molecules into the hollow cavity. Here, AZM was selected to be loaded into the HMSN by a soaking method, yielding HMSN-AZM. The loading capacity of AZM calculated from the UV-vis absorption standard curve was proved to be about 12.5 w/w% (**Figure S4**). Then, GOX was modified on the surface of HMSN-AZM to obtain GOX-HMSN-AZM, endowing the nanoparticles with the catalytic ability. As shown in **Figure 1B,** AZM loading and GOX modification do not affect the structure and morphology of the nanoparticles. The high-angle annular dark-field (HAADF) STEM image of GOX-HMSN-AZM in **Figure 1C** also shows a clear hollow nanostructure, where the distribution of three major elements (Si, O, and N) is uniform (**Figure 1D**). The hydrodynamic diameter of the nanoparticles also shows negligible changes after loading and modification, indicating the high dispersity of the nanoparticles (**Figure 1E**). As shown in **Figure 1F**, the zeta potential of nanoparticles transformed from -23.3 mV to 27.8 mV after loading and modification, which demonstrated the successful preparation of GOX-HMSN-AZM. The X-ray diffraction (XRD) patterns (**Figure 1G, Figure S5A**) further confirm that AZM had been successfully encapsulated into the HMSN. Raman spectroscopic investigation was also conducted. The characteristic peaks of AZM appear at 1450 cm^-1^ and 2934 cm^-1^ (**Figure 1H, Figure S5B**), which demonstrates the successful loading of AZM. UV-vis spectra confirmed that GOX has been successfully combined on the surface of HMSN-AZM (**Figure 1I**). FTIR analysis was further carried out to verify the modification of GOX (**Figure 1J**). The new peak at 1537 cm^-1^ is corresponding to the characteristic peak of GOX (**Figure S5C**). Altogether, the results above indicate that we have successfully designed and prepared monodisperse GOX-HMSN-AZM nanoparticles.

### Catalytic activity by GOX-HMSN-AZM

**Figure 2:**
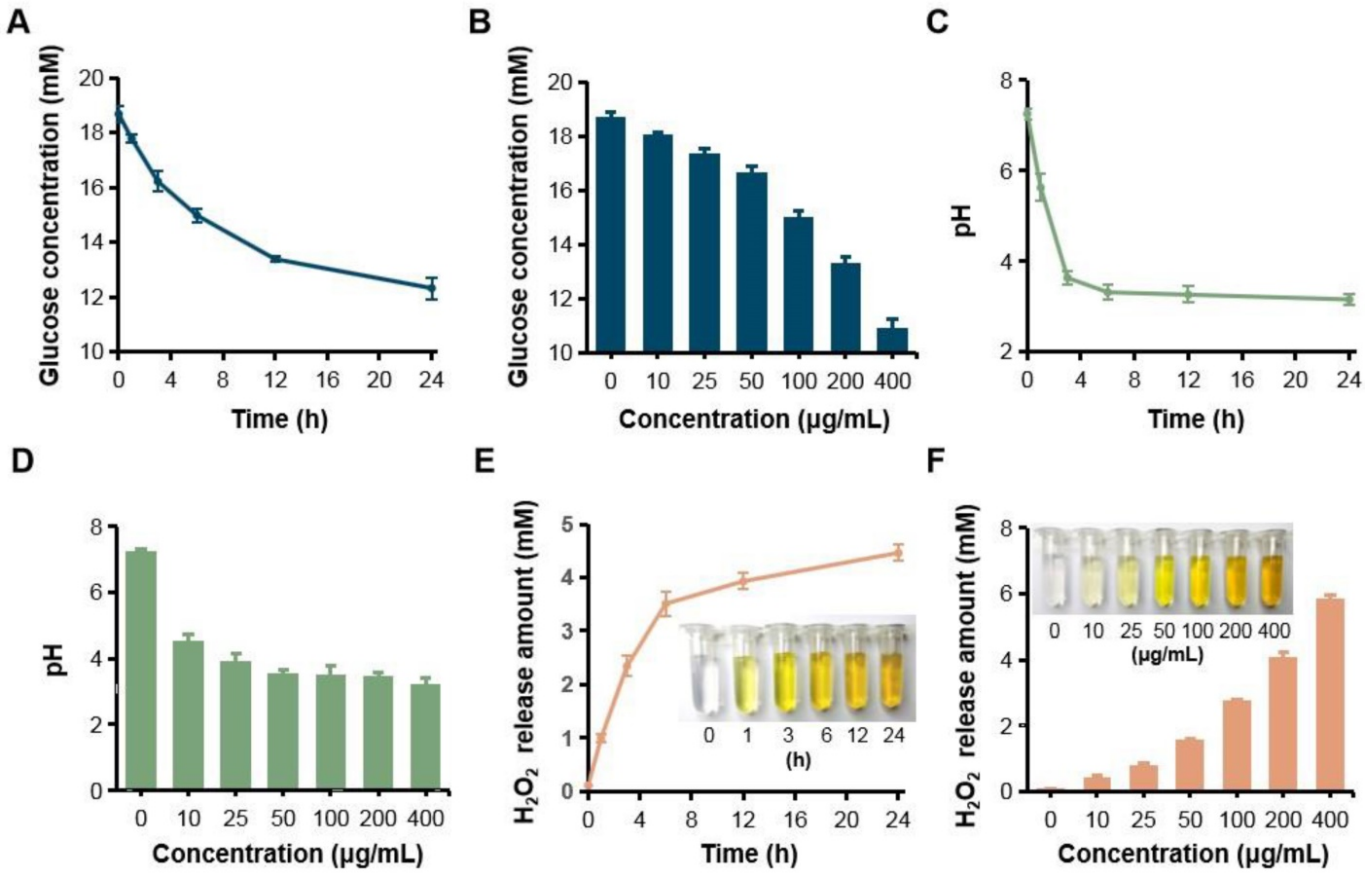
** H_2_O_2_ generation, glucose consumption and pH decrease by GOX-HMSN-AZM.** (A) The glucose concentration variation under the catalyst of 200 μg/mL GOX-HMSN-AZM on different reaction times. (B) The glucose concentration under the catalyst of different concentrations of GOX-HMSN-AZM for 12 h. (C) pH variation on different reaction times under the catalyst of 200 μg/mL GOX-HMSN-AZM. (D) pH variation of glucose solution catalyzed by different concentrations of GOX-HMSN-AZM for 3h. (E) The concentration of generated H_2_O_2_ catalyzed by 200 μg/mL GOX-HMSN-AZM on different reaction times. (F) The concentration of generated H_2_O_2_ catalyzed by different concentrations of GOX-HMSN-AZM for 12 h.

Since GOX can specially catalyze glucose oxidation into gluconic acid and H_2_O_2_, the catalytic capacity of GOX-HMSN-AZM was evaluated by measuring the glucose consumption, gluconic acid production and H_2_O_2_ production. Firstly, the consumption of glucose was evaluated during the reaction by a glucometer. With the accumulation of time, glucose which was incubated with GOX-HMSN-AZM at 200 μg/mL gradually reduced (**Figure 2A**). At the same reaction time for 12 h, glucose concentration reduced along with the increasing concentrations of the GOX-HMSN-AZM nanoparticles (**Figure 2B**). Then, the gluconic acid production was assessed by the detection of pH variation. The solution changed from neutral (~7.26) to acidic (~3.15) within 24 hours (**Figure 2C**). The pH value declined quickly in the first three hours and then changed tardily. At a fixed reaction time for 12 h, the pH of the solution decreased along with the GOX-HMSN-AZM concentration increased (**Figure 2D**). Subsequently, the titanium sulfate colorimetric method 49 was employed to test the generation of H_2_O_2_ (**Figure S6**). Under the catalyst of 200 μg/mL GOX-HMSN-AZM, H_2_O_2_ is produced sustainably over time (**Figure 2E, Figure S7A**). As shown in **Figure 2F** and **Figure S7B**, the generated H_2_O_2_ was increased in response to the elevated concentrations of GOX-HMSN-AZM which was incubated with glucose for 12 hours.

In order to further understand the reactivity of the encapsulated enzymes, we evaluated the kinetic parameters utilizing the GOX-HMSN and GOX-HMSN-AZM (**Table S1**). K_m_ value represents the binding affinity of the enzyme to the substrate and K_cat_/K_m_ value is defined as the catalytic efficiency of enzymatic reaction. Therefore, owing to the spatial separation, the loading of AZM did not interfere with the activity of GOX, leading to the equivalent catalytic efficiency between GOX-HMSN and GOX-HMSN-AZM. The storage stability of both GOX-HMSN and GOX-HMSN-AZM was also evaluated. After 30-day storage at 4 ºC, GOX-HMSN and GOX-HMSN-AZM retained 73.9% and 70.4% of initial enzymatic activity respectively (**Figure S8**).

Furthermore, *in vitro* release study of AZM was also conducted in the research. As shown in **Figure S9**, in the presence of 20 mM glucose, the release rate of the GOX-HMSN-AZM showed a 6.1-fold increase in comparison to the HMSN-AZM. The significant increase in AZM release efficiency in the GOX-HMSN-AZM group mainly benefits from the decline of pH caused by the gluconic acid generated by the GOX catalytic reaction. The above results indicated that GOX-HMSN-AZM effectively catalyzed glucose to reduce local glucose concentration, generate H_2_O_2_ and promote the release of AZM, which was expected to improve the environment of diabetic wounds.

### *In vitro* antibacterial activity of GOX-HMSN-AZM

**Figure 3:**
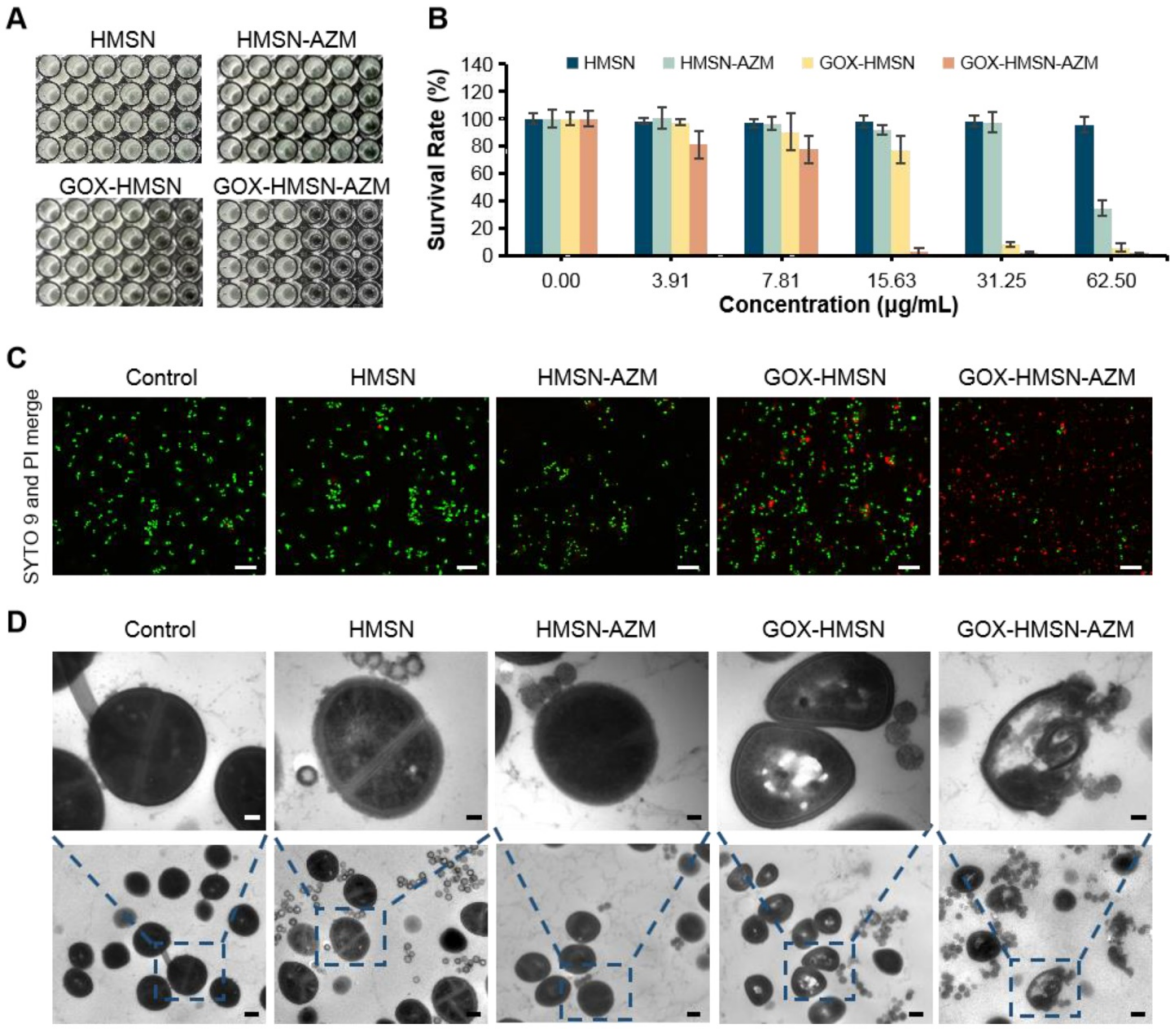
***In vitro* antibacterial activity of GOX-HMSN-AZM.** (A) Planktonic cultivation turbidity (*S. aureus*) with 0, 3.91, 7.81, 15.63, 31.25, 62.50 μg/mL of HMSN, HMSN-AZM, GOX-HMSN and GOX-HMSN-AZM after standard incubation for 24 h. (B) The corresponding survival rate of *S. aureus* incubated with different concentrations of HMSN, HMSN-AZM, GOX-HMSN and GOX-HMSN-AZM after 24 h. (C) Live/dead staining assay of *S. aureus* incubated with PBS, HMSN, HMSN-AZM, GOX-HMSN and GOX-HMSN-AZM by confocal fluorescence microscopy. Live bacteria were stained with SYTO9 (green) and dead bacteria were stained with PI (red). Scale bar = 20 μm. (D) TEM micrographs of *S. aureus* incubated with PBS, HMSN, HMSN-AZM, GOX-HMSN and GOX-HMSN-AZM. Up bar = 0.1 μm, bottom bar = 0.5 μm.

Due to the declined ability to metabolize glucose, the wound conditions of diabetic patients tend to be complicated 3. The characteristic of chronic diabetic wounds is prolonged inflammation and recurrent bacterial infection 23. Staphylococcus aureus (*S. aureus*) infection is reported as the commonest bacterial infection in diabetic wounds 9,50. Thus, in order to evaluate the synergistic antibacterial effect of the nanocomposites, the survival assay, live/dead staining and bacterial morphology were performed in* S. aureus*. As shown in **Figure 3A-B,** the bacterial survival rate of HMSN treaded group showed no obvious difference with the untreated control group, indicating the non-toxicity of HMSN as the nanocarrier. Due to the low water solubility, free AZM demonstrated poor antibacterial efficacy (**Figure S10**). It is worth noting that, with the nanoparticle concentration increases, HMAN-AZM showed a limited antimicrobial effect in high glucose environment. Meanwhile, GOX-HMSN showed improved antimicrobial activity, but it was still limited at low concentrations. However, GOX-HMSN-AZM showed significant improvement in antibacterial capability and could significantly reduce the number of viable bacteria at a low concentration of 15.63 μg/mL (**Figure 3B**). Furthermore, the bacterial resistance test was occupied. As shown in **Figure S11**, the MIC value of *S. aureus* incubated with free AZM increased 80-fold after 10 passages incubation, indicating serious drug resistance. Notably, the MIC value of *S. aureus* incubated with free GOX-HMSN-AZM increased 16-fold compared to passage 1.

Then, the SYTO9/PI live/dead fluorescent staining assay was employed in the current study to analyze the survival of *S. aureus* with different treatments. As shown in **Figure 3C**, the fluorescence intensities of SYTO9 (green) and PI (red) in all the five groups were consistent with the bacterial survival assay results. Scarcely any bacteria stained by PI (red fluorescence) could be observed in control, HMSN treated groups. Compare with the HMSN-AZM and GOX-HMSN groups, an increased red fluorescence could be observed in GOX-HMSN-AZM group, which demonstrated the rapid antibacterial efficiency of GOX-HMSN-AZM nanoparticles in the hyperglycemia environment.

To further study the antibacterial process of the GOX-HMSN-AZM nanoparticles, the structure variation of the bacteria was employed by TEM. As shown in **Figure 3D**, the cytoplasm of the bacteria incubated with GOX-HMSN and GOX-HMSN-AZM partially formed aggregations, indicating the damage of the cells. It is worth noting that the bacterial membranes were most lysed after GOX-HMSN-AZM treatments, leading to the loss of structural integrity of bacterial cell walls. Taken together, these results suggested the strong interaction between GOX-HMSN-AZM and bacteria by disrupting the bacteria cell wall and membrane integrity, indicating the remarkably improved synergistic therapeutic effects of antibiotics and enzymatic reactions, much better than either single treatment alone.

### Antibiofilm Activity of GOX-HMSN-AZM

Traditional antibiotic treatment is often less effective in diabetics owing to the presence of bacterial biofilms in the wound 51. Biofilm has been recognized as the dominant mode of bacterial growth in nature 21. It constitutes a protected mode of growth through quorum sensing and exhibits an increased tolerance to antimicrobial agents, leading to the failure of antibiotic treatments 52-55. Consequently, the antibiofilm activity of composite nanoparticles was evaluated in the study.

**Figure 4:**
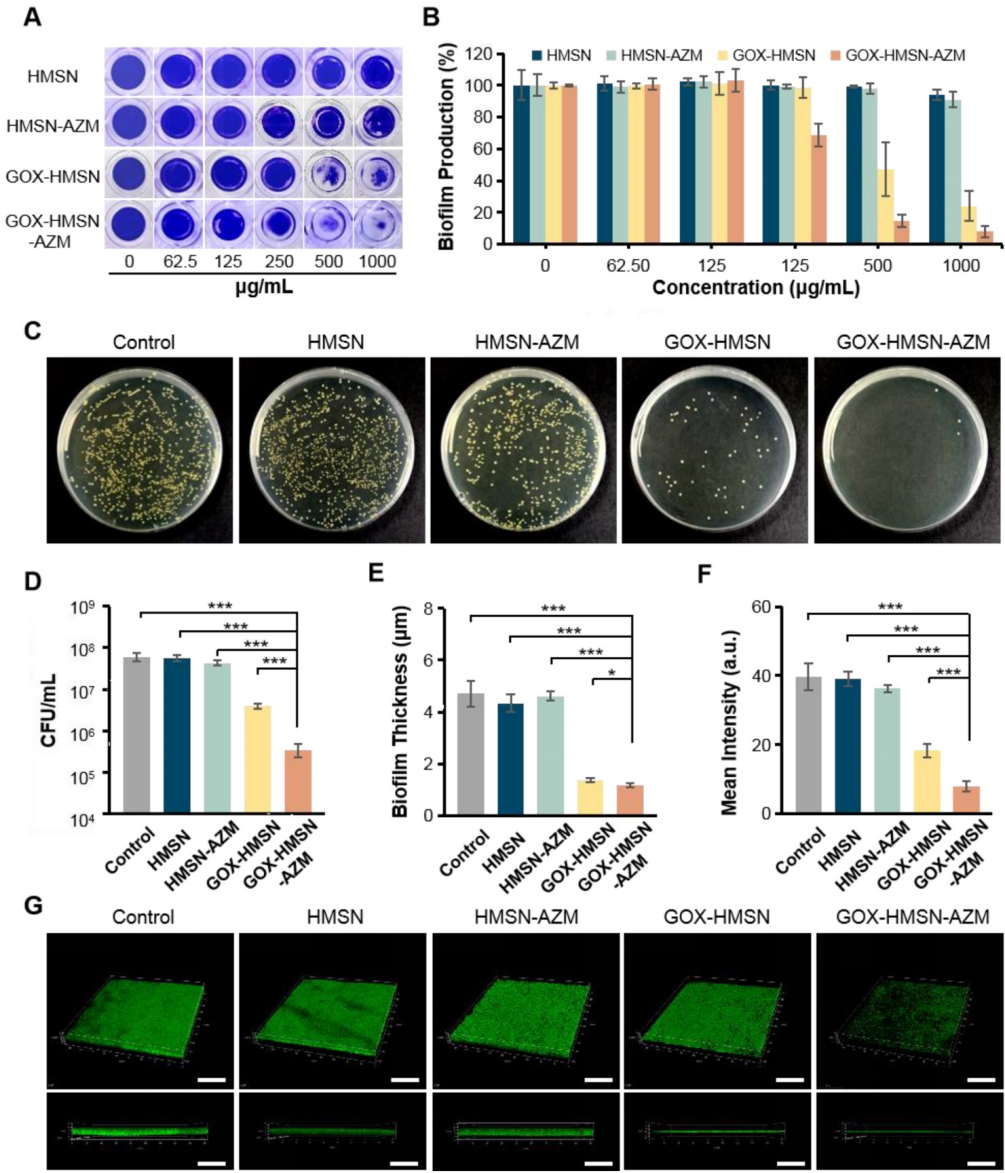
** Antibiofilm Activity of GOX-HMSN-AZM.** (A) Photographs of crystal violet-stained biofilms after different treatments. (B) The corresponding eradication effect of different treatments against *S. aureus* biofilms. (C) Photographs of bacterial colonies formed by *S. aureus* in biofilms which were treated with 500 μg/mL of PBS, HMSN, HMSN-AZM, GOX-HMSN and GOX-HMSN-AZM. (D) The corresponding colony forming unit (CFU) count of *S. aureus* in biofilms with different treatments. (E) The measurement of biofilm thickness. (F) The relative fluorescence intensity of biofilms after different treatments. (G) Three-dimensional confocal fluorescence microscopy images of *S. aureus* biofilms after different treatments. Live bacteria were stained with SYTO 9 (green fluorescence). Scale bar = 50 μm. (*p < 0.05, **p < 0.01, ***p < 0.001).

The disruption effect of different treatments against *S. aureus* biofilms was evaluated by crystal violet staining assay. As shown in **Figure 4A**, after treatment with the HMSN and HMSN-AZM, the biofilm structure exhibited negligible changes at tested doses. However, significant biofilm destructions were generated with the treatments of GOX-HMSN and GOX-HMSN-AZM at high concentrations (500 μg/mL). It is worth noting that GOX-HMSN-AZM exhibit a much stronger biofilm destruction capability than the GOX-HMSN even at the same concentrations. The corresponding statistic results in **Figure 4B** and **Figure S12** indicate the percentage of biofilm remaining after treatments. There was no significant difference in the biofilm mass residuals treated with HMSN and HMSN-AZM compared to the control. At the concentrations of 500 and 1000 μg/mL, GOX-HMSN-AZM destroyed around 85.3% and 92.1% of the biofilms, which was significantly higher than that with the treatment of GOX-HMSN.

Then the dilution plate assay was employed to further quantify the disruption of different treatments against *S. aureus* biofilm. As shown in **Figure 4C-D**, the quantified results showed that when bacteria were treated with HMSN, there is no significant difference between the number of colonies compared to that of the control. Due to the inherent high antibiotic resistance of biofilms, only 28.6% of bacteria were killed after biofilms were treated with HMSN-AZM at 500 μg/mL. GOX-HMSN showed relatively efficient antibacterial ability, which eliminated 93.3% of *S. aureus* in biofilms. Notably, GOX-HMSN-AZM exhibited better antibacterial ability against *S. aureus* biofilms, which caused a 99.4% reduction of bacterial survival rate at the same concentration.

Ultimately, confocal microscopy was applied to further verify the destruction of *S. aureus* biofilm after different treatments through intuitively observing the variation of biofilm thickness and density. As shown in **Figure 4E**-**G,** the structure of the bacterial biofilms treated with HMSN and HMSN-AZM was intact and compact, which was similar to the control. The treatment of GOX-HMSN eradicated the bacterial biofilm to some extent. In comparison, the bacterial biofilms treated with GOX-HMSN-AZM were thinnest and sparsest among all. Quantitative analysis demonstrated that the thickness of the bacterial biofilms treated with GOX-HMSN-AZM reduced 75.2% and the relative fluorescence intensity reduced 79.8% in comparison to the control group (**Figure 4E-F**), which demonstrated the efficient eradication induced by our strategy. The results above demonstrated that GOX-HMSN-AZM effectively eradicated bacterial biofilm, which was beneficial for treating bacterial infections in chronic diabetic wounds.

### Promoting healing efficacy on diabetic cutaneous wound model

**Figure 5:**
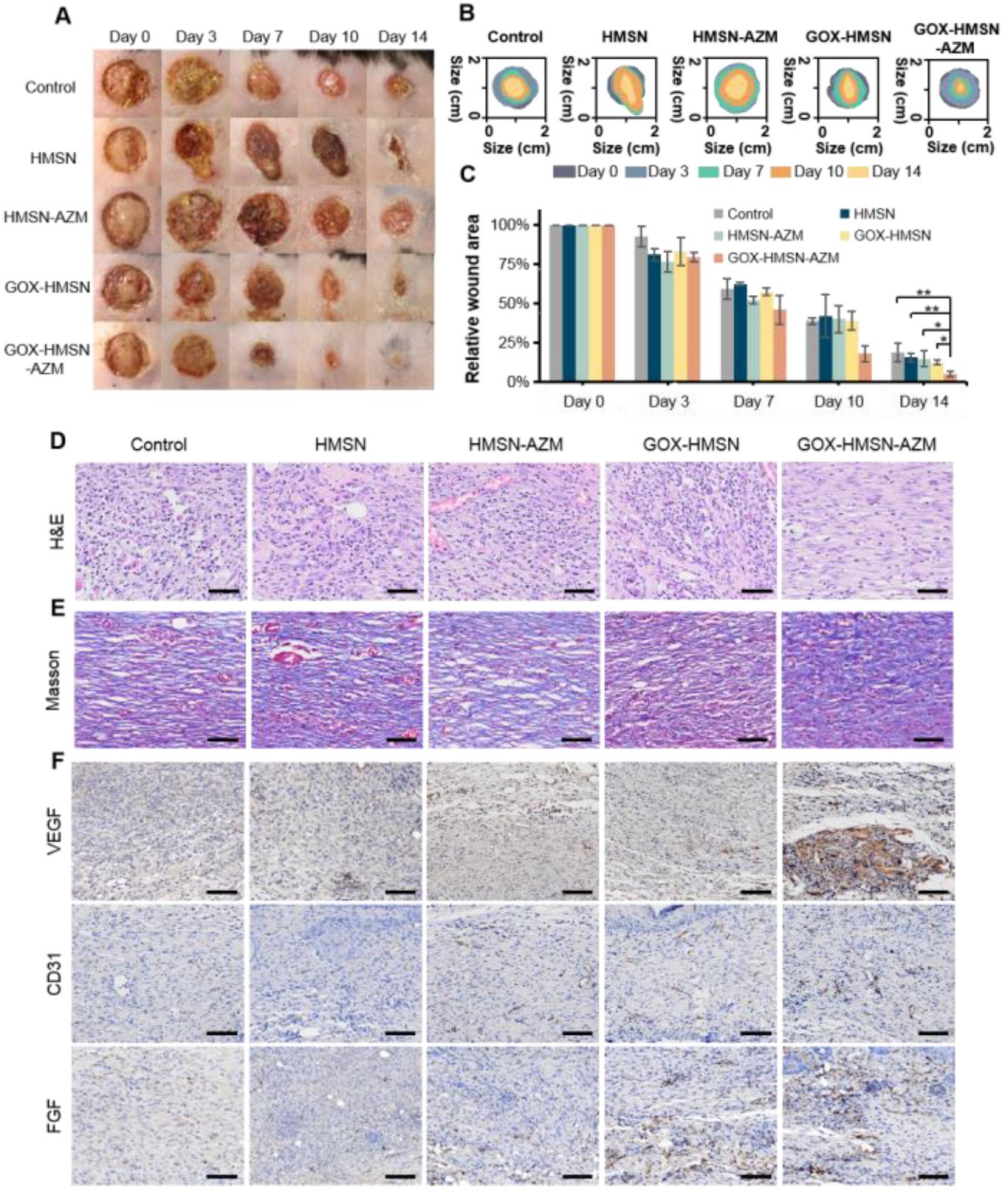
** Promoting healing efficacy on diabetic cutaneous wound model.** (A) The representative photographs of the *S. aureus* infectious wound on db/db mice after different treatments. (B) Traces of wound healing process of mice after different treatments. (C) Quantification of the percentage of wound healing area at different time points. (D) H&E staining, (E) Masson's trichrome staining and (F) immunohistochemical staining of VEGF, CD31 and FGF of the db/db mice dermal wound tissue at day 14 after treatments. Scale bar = 100 μm. (*p < 0.05, **p < 0.01, ***p < 0.001).

Due to the complicated physiological conditions related to hyperglycemia, diabetic wounds generate prolonged inflammation, recurrent infections which greatly extend the treatment period 23. On the basis of *in vitro* results, we further verified whether the composite nanoparticles can accelerate the wound healing process in diabetic infectious wounds. Thus, the bacteria-infected cutaneous wound model in diabetes (db/db) mice was used to evaluate the antibacterial effects and wound healing effects of our strategy. The treatments were applied 24 hours after infection, when the bacterial biofilms were formed in the wound areas (**Figure S13**). Representative pictures of the infected wounds were shown in **Figure 5A** and the wound areas were also measured (**Figure 5B-C**). The wounds of mice treated with GOX-HMSN-AZM almost removed the infection and healed the wounds after 14 days, whereas other treatments induced incomplete recovery. The HMSN-AZM and GOX-HMSN groups had a therapeutic effect on diabetic wounds to some extent. However, due to the limited therapeutic effect of individual treatments, it could be observed the invasive and persistent bacterial infection in the surrounding area around the wound in the diabetic mice in the group of HMSN-AZM, and GOX-HMSN. It is noteworthy that the diabetic wounds treated with GOX-HMSN-AZM exhibited speedy and efficacious recovery due to the high antibacterial and antibiofilm activity of GOX-HMSN-AZM in hyperglycemia environment.

Subsequently, the wound epithelial tissue was excised and stained for histological analysis. Hematoxylin and eosin-stained section (H&E) demonstrated the inflammation status of the wound (**Figure 5D**). For the untreated group, the histological slice displayed the obvious infiltration of inflammatory cells, indicating an intense immune response against bacterial infection. In contrast, fewer inflammatory cells were observed in the group treated with GOX-HMSN-AZM, which demonstrated the effective antimicrobial and infection suppression of the treatment strategy. Moreover, the collagen fibers in the wound tissue treated with GOX-HMSN-AZM were dense and ordered, indicating superior wound repair in comparison to other treatment groups (**Figure 5E**).

To evaluate the mechanism of GOX-HMSN-AZM in promoting wound healing, tissue slices collected from different groups were analyzed by immunohistochemistry. It is generally acknowledged that the process of angiogenesis is vital for optimal wound repair 56, 57. Thus, vascular endothelial growth factor (VEGF), platelet endothelial cell adhesion molecule-1 (CD31) and fibroblast growth factor (FGF), were assessed after treatments. VEGF is an inducer of angiogenesis, which is considered to be a crucial regulator of vasculogenesis 58, 59. As shown in **Figure 5F** and **Figure S14**, VEGF was upregulated with the treatment of GOX-HMSN-AZM, showing a 5.71-fold increase in comparison to the control group. Additionally, microvessel density (MVD) was assessed using immunohistochemical staining of CD31 60. Compared with the other 4 groups, there was a significant improvement of the total CD31 expression in the GOX-HMSN-AZM group, which revealed the well-formation of blood vessels by the treated of GOX-HMSN-AZM. FGF activates receptors usually indirectly stimulate angiogenesis by inducing the release of angiogenic factors from other cell types 61. As shown in **Figure 5F** and **Figure S14**, FGF was also significantly upregulated in GOX-HMSN-AZM group. Taken together, these results demonstrated that GOX-HMSN-AZM could accelerate the wound healing process of bacteria-infected diabetic mice by reducing the burden of bacterial infection and promoting the formation of more vessels.

**Figure 6:**
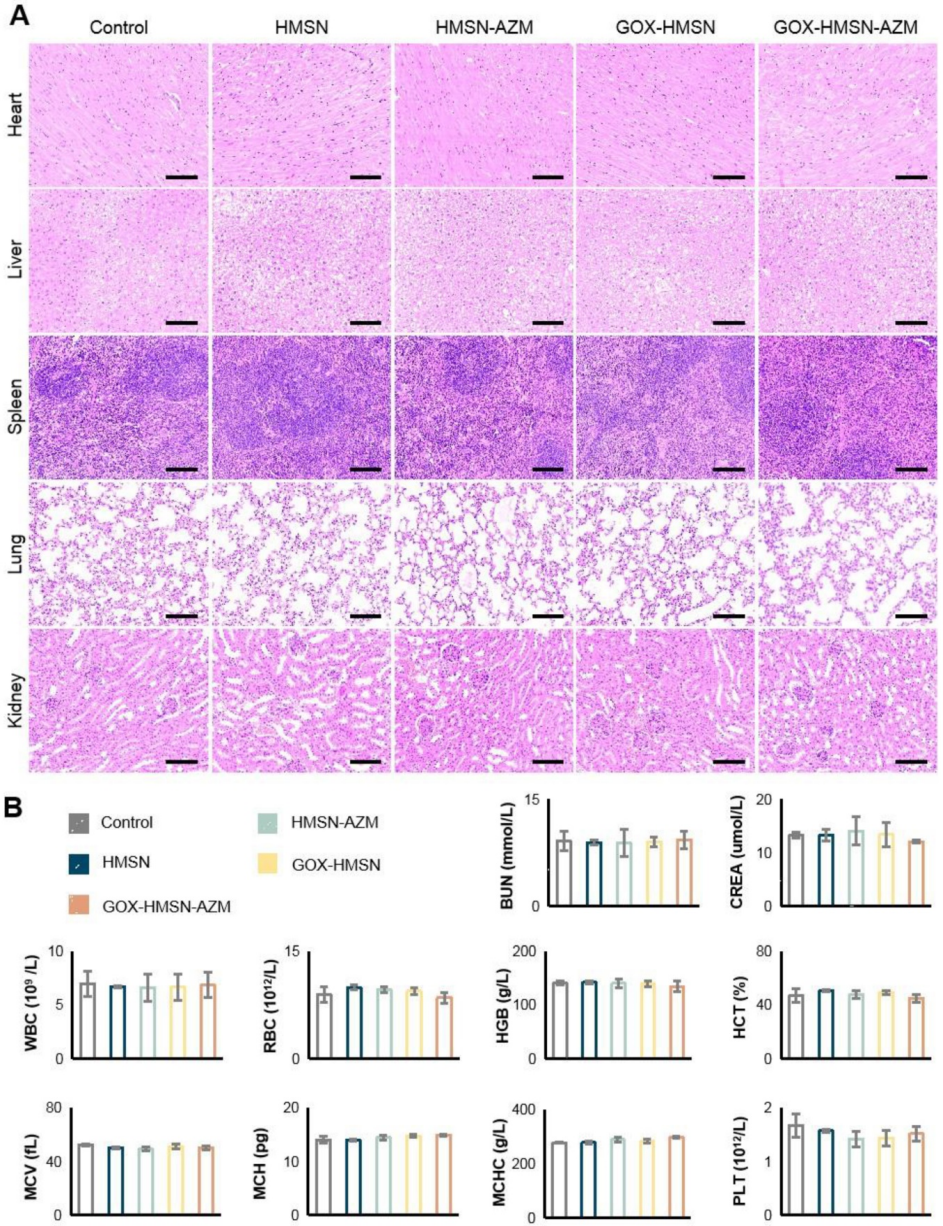
** Preliminary toxicity study.** (A) H&E staining of the major organs of the mice at 14 days after different treatments. Scale bar = 100 μm. (B) Blood biochemistry and hematology examination of the db/db mice 14 days after treatments. Blood biochemistry test. BUN, blood urea nitrogen; CREA, creatinine. Hematology examination. WBC, white blood cells; RBC, red blood cells; HGB, hemoglobin; HCT, hematocrit; MCV, mean cell volume; MCH, mean corpuscular hemoglobin; MCHC, mean corpuscular hemoglobin concentration; PLT, blood platelet.

The safety of the treatments was further assessed. During the entire therapy period, the body weight of the treated mice was stable and there were no significant differences between the groups (**Figure S15**). **Figure 6A** showed no noticeable organ damage or inflammatory lesions in histology analysis between each group of mice. Besides, there were no significant changes in blood biochemistry and hematology examination (**Figure 6B**). These results above demonstrated the safety and high biocompatibility of GOX-HMSN-AZM* in vivo*, showing the great potential for our strategy in future clinical translation.

## Conclusion

In summary, we successfully developed multifunctional GOX-HMSN-AZM nanoparticles for the treatment of chronic diabetic wounds. Owing to the unique catalytic capability of GOX, GOX-HMSN-AZM could effectively ameliorate the hyperglycemia environment around the wound and produce H_2_O_2_, simultaneously. The increased acidity by the catalytic reaction also promotes the release of AZM. In the bacteria-infected diabetic cutaneous wound models, the reduction of glucose, generation of H_2_O_2_, and release of AZM could effectively reduce the bacterial infection and promote the wounds healing. This accelerated anti-bacteria and wound healing process are mainly due to the destruction of bacterial biofilm and the acceleration of vascular regeneration by GOX-HMSN-AZM. More importantly, the biosafety of the composite nanoparticles was verified during the therapy. This work provides a meaningful inspiration for the rational design of intelligent nanocomposites to realize efficient therapeutic modalities for chronic diabetic wounds, which has profound significance for clinical application.

## Supplementary Material

Supplementary figures and table.

## Abbreviations

AZM: azithromycin
BET: Brunner-Emmet-Teller
CD31: platelet endothelial cell adhesion molecule-1
FGF: fibroblast growth factor
GOX: glucose oxidase
H_2_O_2_: hydrogen peroxide
H&E: hematoxylin and eosin-stained section
HAADF: high-angle annular dark-field
HMSN: hollow mesoporous silica nanoparticles
MVD: microvessel density
XRD: X-ray diffraction
TEM: transmission electron microscope
VEGF: vascular endothelial growth factor
