# Supplementary material for: Wound microenvironment-responsive glucose consumption and hydrogen peroxide generation synergistic with azithromycin for diabetic wounds healing
Source: Theranostics. 2022 Mar 6;12(6):2658–73. doi: 10.7150/thno.64244 (PMC8965477; doi:10.7150/thno.64244)
Supplement: Supplementary file 1 — Supplementary figures and table. [file thnov12p2658s1.pdf]

Supporting Information

Wound microenvironment-responsive glucose consumption and hydrogen peroxide generation synergistic with azithromycin for diabetic wounds healing

Minqi Shi[1,2], Zhen Du[1,3*], Yuchen Qi[2], Wanlin Li[2], Huiqun Hu[5], Xiuhui Lin[5], Shoujie Wang[1,*], Zhe Tang[1,*] and Min Zhou[1,2,4,*]

[1] Department of Surgery, The Fourth Affiliated Hospital, Zhejiang University School of Medicine, Yiwu 322000, China.

[2] Institute of Translational Medicine, Zhejiang University, Hangzhou 310009, China.

[3] The Cancer Hospital of the University of Chinese Academy of Sciences (Zhejiang Cancer Hospital), Institute of Basic Medicine and Cancer (IBMC), Chinese Academy of Sciences, Hangzhou, Zhejiang 310022, China

[4] State Key Laboratory of Modern Optical Instrumentations, Zhejiang University, Hangzhou 310058, China.

[5] Department of Infectious Diseases, The Second Affiliated Hospital, Zhejiang University School of Medicine, Hangzhou 310009, China.

* Corresponding authors.

E-mail: zhoum@zju.edu.cn (MZ)

8xi@zju.edu.cn (Z.T)

duzhen90@zju.edu.cn (Z.D)

wangshoujie@zju.edu.cn (S.W)

Supplementary Table

Table S1. The kinetic parameters of the different samples using glucose as substrate.

	K_m (mM)	K_{cat} (s^{-1})	K_{cat}/K_m (s^{-1} mM^{-1})
GOX-HMSN	39.64±3.17	499.23±20.89	12.67±0.73
GOX-HMSN-AZM	41.70±5.76	513.62±43.19	12.41±0.80

Supplementary Figures

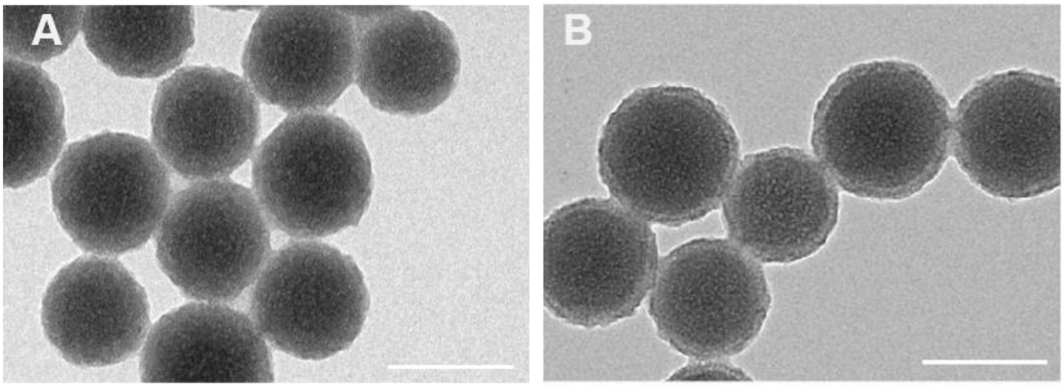

Figure S1. (A) TEM image of $dSiO_2$. (B) TEM image of $dSiO_2$@MSN. Scale bar = 100 nm.

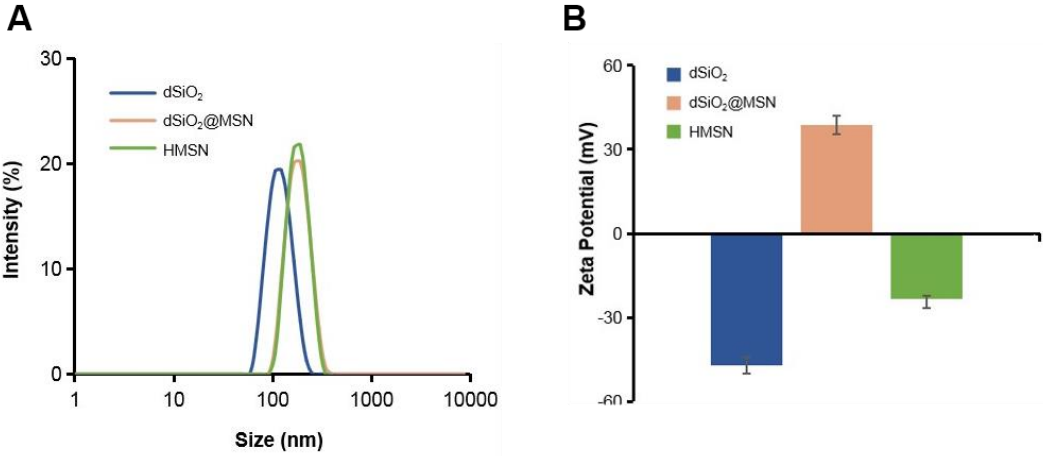

Figure S2. (A) Hydrodynamic diameters of $dSiO_2$, $dSiO_2$@MSN and HMSN, respectively. (B) Zeta potential of $dSiO_2$, $dSiO_2$@MSN and HMSN, respectively.

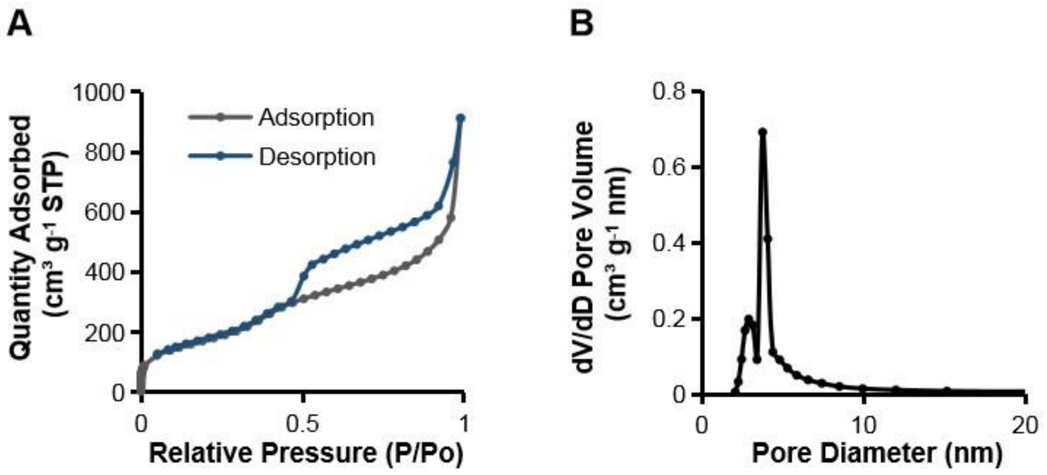

Figure S3. (A) The nitrogen adsorption-desorption isotherm of HMSN. (B) Corresponding pore size distribution of HMSN.

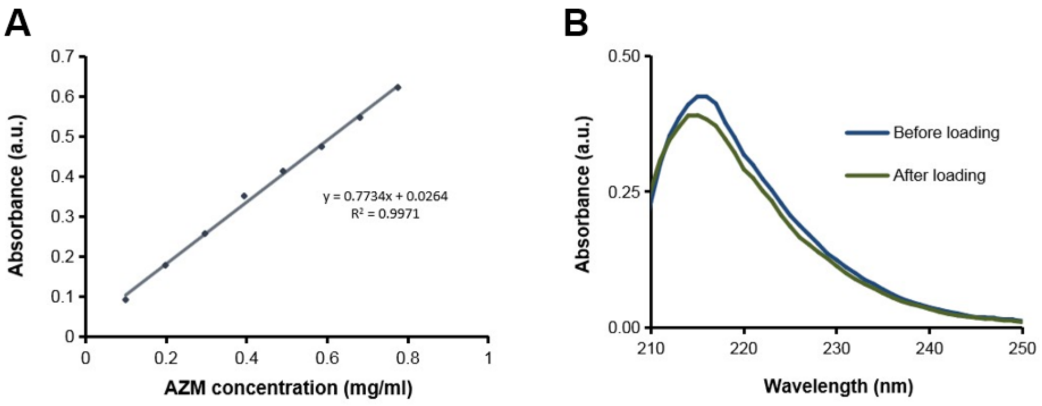

Figure S4. (A) UV-vis absorption standard curve of AZM at 215 nm. (B) UV-vis absorption spectra of the supernatant before and after azithromycin loading to HMSN.

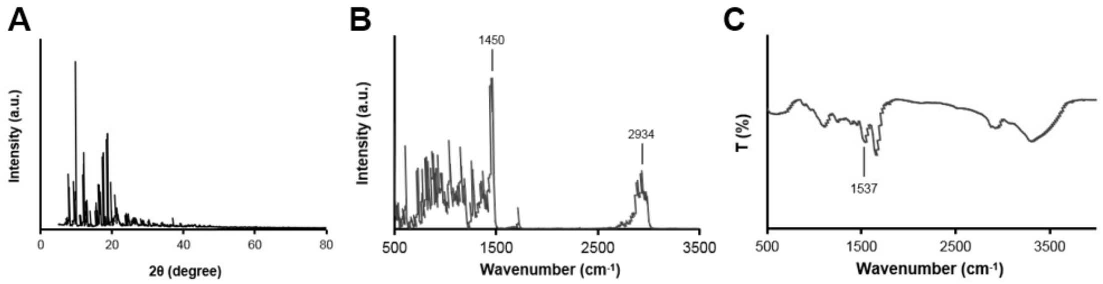

Figure S5. (A) Typical XRD patterns of azithromycin. (B) Raman spectra of azithromycin. (C) FTIR spectra of glucose oxidase (GOX).

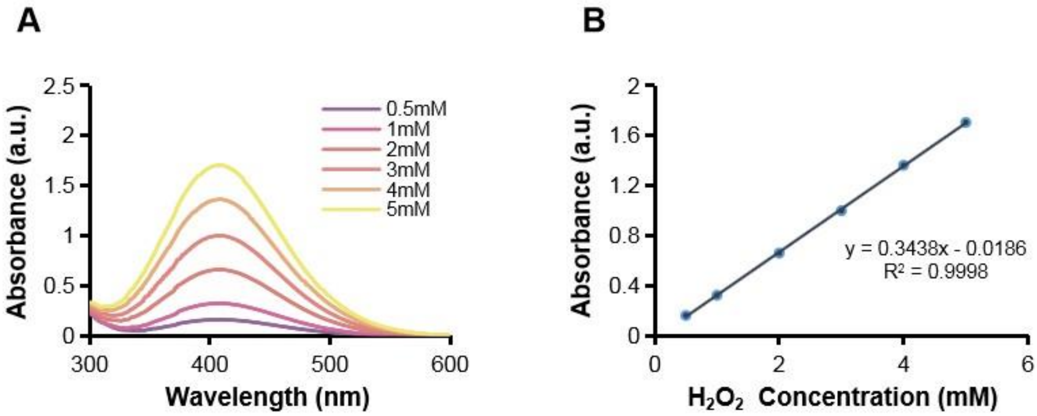

Figure S6. (A) The UV-vis spectra of different concentrations of H_2O_2 using titanium sulfate colorimetric method. (B) The corresponding UV-vis absorption standard curve of H_2O_2 at 405 nm.

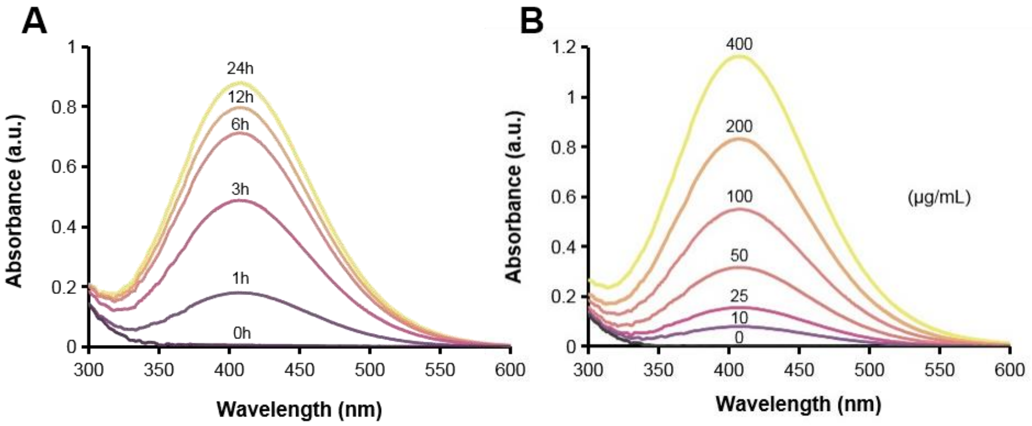

Figure S7. (A) Under the amount of 200 μg/mL GOX-HMSN-AZM, the UV-vis spectra of the generated H_2O_2 concentration on different reaction times by titanium sulfate colorimetric method. (B) The UV-vis spectra of the generated H_2O_2 concentration catalyzed by different concentrations of GOX-HMSN-AZM at 12 h using titanium sulfate colorimetric method.

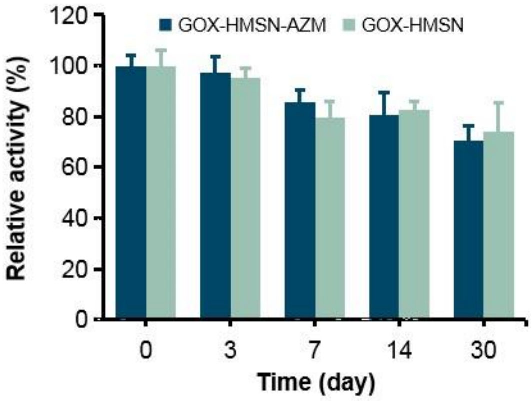

Figure S8. The relative enzymatic activity of GOX-HMSN-AZM and GOX-HMSN stored at 4 °C after 0, 3, 7, 14, and 30 days.

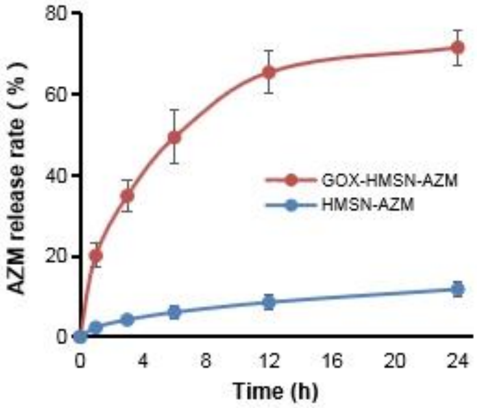

Figure S9. Quantitative analysis of the released azithromycin from HMSN-AZM and GOX-HMSN-AZM.

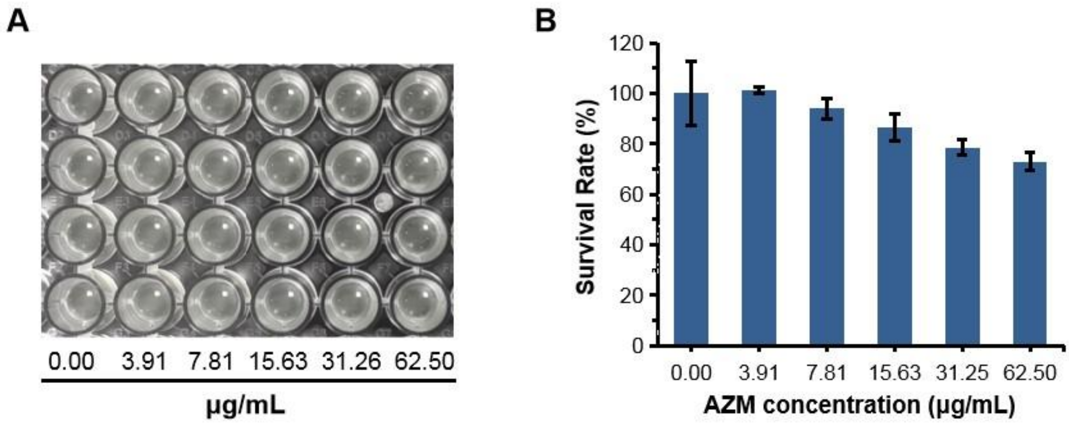

Figure S10. (A) Planktonic cultivation turbidity (*S. aureus*) with 0, 3.91, 7.81, 15.63, 31.25, and 62.50 µg/mL of AZM after standard incubation for 24 h. (B) The corresponding survival rate of *S. aureus* incubated with different concentrations of AZM after 24 h.

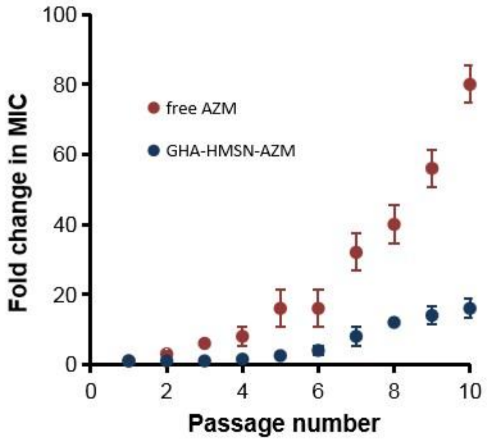

Figure S11. Resistance development of *S. aureus* after continuous exposure toward free AZM or GOX-HMSN-AZM for 10 passages.

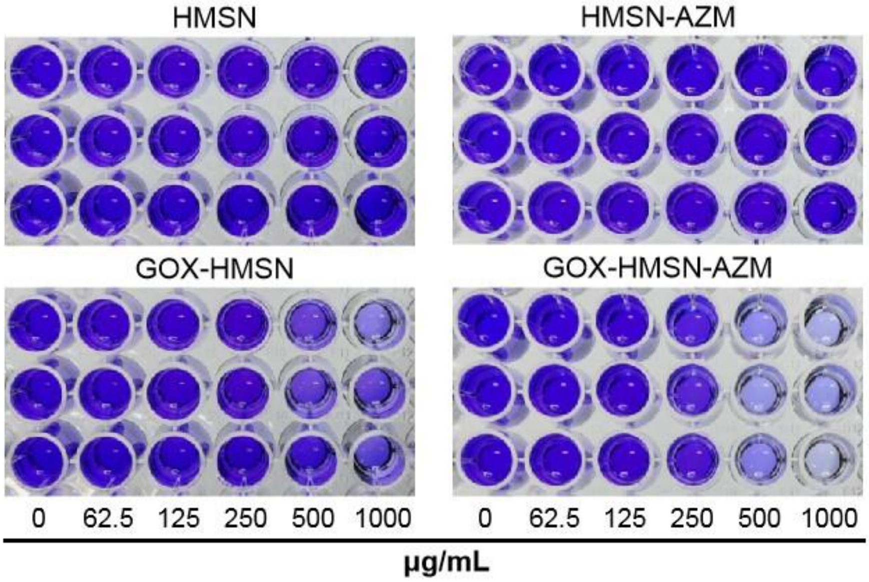

Figure S12. The images of crystal violet staining assay. The optical densities of the wells represent biofilm mass residuals.

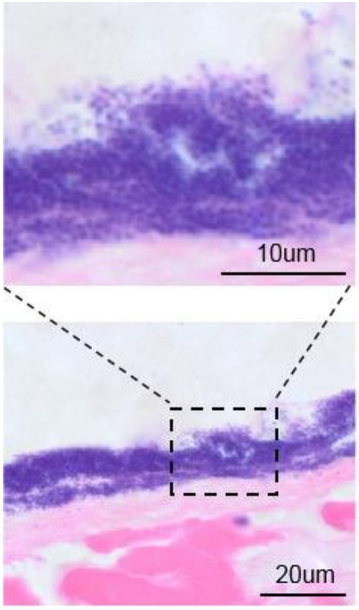

Figure S13. Micrographs of H&E staining of the db/db mice dermal wound tissue after inoculation with *S. aureus*.

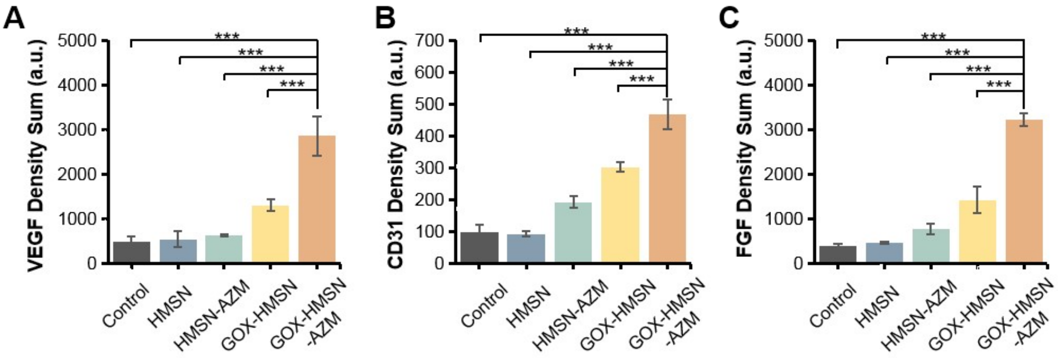

Figure S14. Corresponding quantitative analysis of VEGF, CD31 and FGF in immunohistochemical assay. (*$p < 0.05$, **$p < 0.01$, ***$p < 0.001$).

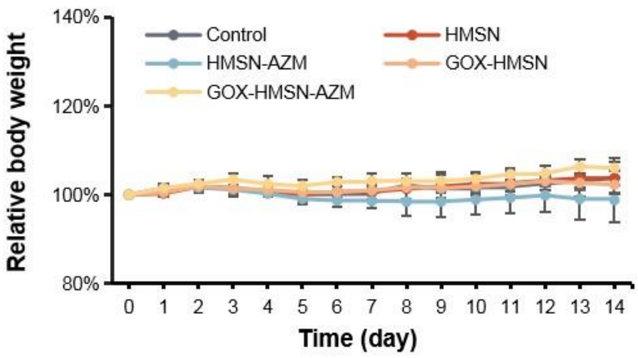

Figure S15. Weight change curves of mice after different treatments. There were negligible changes during 14 days, indicating the relatively high safety of these treatments.
